# Genomic insights into shank and eggshell color in Italian local chickens

**DOI:** 10.1016/j.psj.2024.103677

**Published:** 2024-03-21

**Authors:** Francesco Perini, Filippo Cendron, Emiliano Lasagna, Martino Cassandro, Mauro Penasa

**Affiliations:** ⁎ Department of Agronomy, Food, Natural resources, Animals and Environment, University of Padova, Legnaro, Padua 35020, Italy; † Department of Agricultural, Food and Environmental Sciences, University of Perugia, Perugia 06121, Italy

**Keywords:** native breed, phenotype, pigmentation, SNP, GWAS

## Abstract

Eggshell and shank color in poultry is an intriguing topic of research due to the roles in selection, breed recognition, and environmental adaptation. This study delves into the genomics foundations of shank and eggshell pigmentation in Italian local chickens through genome-wide association studies analysis to uncover the mechanisms governing these phenotypes. To this purpose, 483 animals from 20 local breeds (n = 466) and 2 commercial lines (n = 17) were considered and evaluated for shank and eggshell color. All animals were genotyped using the Affymetrix Axiom 600 K Chicken Genotyping Array. As regards shank color, the most interesting locus was detected on chromosome Z, close to the *TYRP1* gene, known to play a key role in avian pigmentation. Additionally, several novel loci and genes associated with shank pigmentation, skin pigmentation, UV protection, and melanocyte regulation were identified (e.g., *MTAP, CDKN2A, CDKN2B*). In eggshell, fewer significant loci were identified, including *SLC7A11* and *MITF* on chromosomes 4 and 12, respectively, associated with melanocyte processes and pigment synthesis. This comprehensive study shed light on the genetic architecture underlying shank and eggshell color in Italian native chicken breeds, contributing to a better understanding of this phenomenon which plays a role in breed identification and conservation, and has ecological and economic implications.

## INTRODUCTION

Researchers have been fascinated by the vibrant and heterogeneous colors of avian species. The colors and patterns of eggshell and shank have drawn the most interest among the different elements of bird color because they play crucial roles in selection, breed recognition, and environmental adaptability (Lawal and Hanotte, 2021). The genetic basis of shank and eggshell pigmentation in chickens (*Gallus gallus domesticus*) provides an insight into the complex mechanisms underlying these phenotypes. Understanding the genetic basis of pigmentation is not only of biological interest but it has also practical consequences for poultry breeding and conservation as these traits have both ecological and economic importance. Over the past few decades, the insights into the genomic underpinnings of avian pigmentation have evolved substantially (Zheng et al., 2020), and much of this progress can be attributed to the development of advanced molecular biology techniques, high-throughput sequencing, and powerful bioinformatic tools.

The pigmentation of shanks in chickens is a complex trait regulated by several genes and genetic pathways, mainly represented by melanin production (Li et al., 2020). The primary pigments involved in shank colors are eumelanin, which is responsible for black and brown colors, and pheomelanin, which is responsible for red and yellow colors (Zheng et al., 2020). Some of the key genes that have been implicated in the process of melanin production in chicken are Melanocortin 1 Receptor (***MC1R***), Tyrosinase (***TYR***), Agouti Signaling Protein (***ASIP***), Membrane-associated Transporter Protein (***SLC45A2***), Premelanosome Protein (***PMEL***), and Tyrosinase-related Protein 1 (***TYRP1***) (Jin et al., 2014; Yang et al., 2019; Zheng et al., 2020).

Eggshell pigmentation is an economic important and complex trait in poultry species. Eggshell color is determined by a wide range of factors such as the biliverdin and protoporphyrin, which play a central role in determining the green and blue colors, and red-brown color of the eggshell, respectively (Hargitai et al., 2017). Furthermore, eggshell pigmentation is influenced by bird oxidative status level (Duval et al., 2016). As regards the genetic background, a huge number of genes are implicated in eggshell color and the debate is still open because the role of many of them is not clear. Some examples of certain genes involved in eggshell colors are heme oxygenase (decycling) 1 (***HMOX1***) which converts protohaem into biliverdin, and genes linked to biliverdin production like Biliverdin Reductase *(****BLVRA***) and ferrochelatase (***FECH***) which integrates ferrous ions into protoporphyrin (Bai et al., 2019).

A genomic feature related to the role of the genes present in the sexual chromosomes needs to be better investigated. Indeed, it has been demonstrated that the Z chromosome in chicken contains the loci directly or indirectly implicated in shank and eggshell color (Heo et al., 2023).

In the present study the genomic basis of the shank and eggshell pigmentation was explored. For the first time, a genome-wide association study (**GWAS**) analysis on eggshell and shank pigmentation was performed using a high-density 600 K SNP array in Italian local chicken breeds. We delve into the current state of knowledge regarding the genetic and genomic signatures that govern the genes’ expression and pathways, allowing specific phenotype colors.

## MATERIALS AND METHODS

### Ethical Statement

Ethical approval was not required for the current study. Blood samples were collected in compliance with the European rules [Council Regulation (EC) No. 1/2005 and Council Regulation (EC) No. 1099/2009] during routine health controls by the public veterinary service.

### Samples Collection, Genotyping and Data Filtering

Data used in the present study represent a subset of 466 animals of 20 local breeds and 17 animals of 2 commercial lines intended for egg production, extracted from the original data described by Cendron et al. (2020). Briefly, the original dataset included from 20 to 24 animals (equal number of males and females) for each of 23 local breeds and all the animals were blood sampled for DNA extraction. To ensure representativeness of the study, samples were collected from at least 3 distinct farms and/or conservation centers per breed. The DNA extraction was carried out by Neogen (Ayr, Scotland, UK) as well as genotyping through Affymetrix Axiom 600 K Chicken Genotyping Array, representing 580,961 SNPs. The reference genome for genotyping was the *Gallus gallus 6.0* chicken assembly GRCg6a (accession number: GCA_000002315.5), which includes the markers found on chromosomes 1 through 33 and sex chromosomes. The SNPs with call rate <95% and minor allele frequencies <5%, and animals with more than 10% of missing genotypes were discarded from the dataset. Additionally, a sex-based quality check was performed applying the same filtering parameters only to chromosome Z. Furthermore, while the sex of each sample was recorded during the sampling procedure, sex imputation was performed based on the genomic features of the sexual chromosomes and samples that did not match sex values were removed from the dataset. Finally, the heterozygosity level of chromosome Z was assessed in female samples and those with a high level of heterozygosity were excluded (König et al., 2014). All the checks were performed using PLINK software v1.9 (Chang et al., 2015).

### Phenotypes and Population Structure

From the dataset, the breeds of interest grouped according to shank and eggshell pigmentation were extracted. As regards shanks, we used a case population characterized by a phenotype classified as dark, whose animals presented grey-black and dark green shank. The case population was formed by 201 animals (137 females and 64 males) of 9 local breeds, namely Padovana Argentata (**PPA**), Padovana Camosciata (**PPC**), Padovana Dorata (**PPD**), Polverara Bianca (**PPB**), Polverara Nera (**PPN**), Cornuta di Caltanissetta (**COR**), Valplatani (**VAP**), Siciliana (**SIC**), and Romagnola (**ROM**). The reference population was formed by 155 animals (116 females and 39 males) of 7 local breeds with yellow-shank pigmentation, namely Bionda Piemontese (**BIP**), Ermellinata di Rovigo (**PER**), Mericanel della Brianza (**MER**), Pepoi (**PPP**), Robusta Lionata (**PRL**), Robusta Maculata (**PRM**), and Valdarnese Bianca (**VAD**) (Supplementary Table 1). Shank phenotypes were partially described by Perini et al. (2020).

As regards eggshell phenotype, the case population was formed by 127 individuals (96 females and 31 males) of 5 local breeds (BIP, PPP, PRM, PRL, and PER) and 2 commercial lines (Eureka and Isa Brown) with tinted (different brownness levels) eggshell (Cendron et al., 2023). The control population was formed by 277 individuals (199 females and 78 males) of 12 local breeds which typically produce eggs with white eggshell: PPA, PPB, PPC, PPD, VAP, COR, ROM, VAD, Livorno Bianca (**LVB**), Livorno Nera (**LVN**), Modenese (**MOD**), and Ancona (**ANC**).

Principal component analysis (**PCA**) was used to estimate the population structure of the final dataset. First, we extracted the case and the control populations from the .ped file using the PLINK command "–keep", creating 2 subsets for the 2 phenotypes under consideration. Then PLINK software v1.9 was used to calculate the eigenvectors and eigenvalues, and then the ggplot2 (v3.1.0) package of R software was used to plot results of the PCA (Wickham, 2016).

### GWAS

A GWAS was performed to compare the case and control populations for shank and eggshell phenotypes. The GWAS was carried out through the GEMMA software v0.94.128 with linear mixed models, and corrected for the effect of gender and relatedness with the centered relatedness matrix (Zhou and Stephens, 2014). The linear mixed model was applied to each chromosome (including sex Z chromosome), by incorporating random genetic effects with genomic relationships to correct for genetic structure in the chosen population. The Bonferroni approach was used to establish the genome-wide significance threshold, dividing the standard *P*-value (0.05) by the number of tests done as follows: cutoff = −log_10_(0.05/number of variants). In each investigation, the genomic inflation factor (λ) was calculated as the ratio between the median of the resulting chi-squared test statistics and the expected median of the chi-squared distribution. For this purpose, the qqman package of the R software was used, with the median option to evaluate the inflation of the test statistics that could lead to overestimation (Turner, 2018). QQplots and lambda values are reported in Supplementary Figure 3. Variants were then annotated in vCard File (**VCF**) format using snpEFF version 5.1 with default parameters (Cingolani et al., 2012). For further analysis, only SNPs with significant *P*-values were considered. The VCF file was then used as input in Ensembl Variant Predictor (**VEP**) to assess the impact of SNPs on genes, transcripts, and protein sequences. Moreover, the R software package GALLO was used to annotate the significant SNPs to confirm annotation obtained previously from VEP, and to inspect genes around the identified SNPs within a range of 0.1 Mb (Fonseca et al., 2020). The annotation was carried out on the GRCg6a version of reference genomes, as it was built on Red Jungle Fowl, which is more similar to the local breeds here presented than the GCRg7b, a more updated reference genome but built on Broiler genomic features (Smith et al., 2023). The *F*_ST_ values between the experimental and the control populations were calculated with PLINK software v1.9. Among the significant SNPs, 4 with a location inside or close to genes of interest and the lowest *P*-value out from GWAS regarding the shank phenotype, and 3 in eggshell phenotype were detected. The same SNPs were processed to compute the r2 values in order to calculate the Linkage Disequilibrium (**LD**) between selected SNPs and the genomic region.

## RESULTS

**Figure 1:**
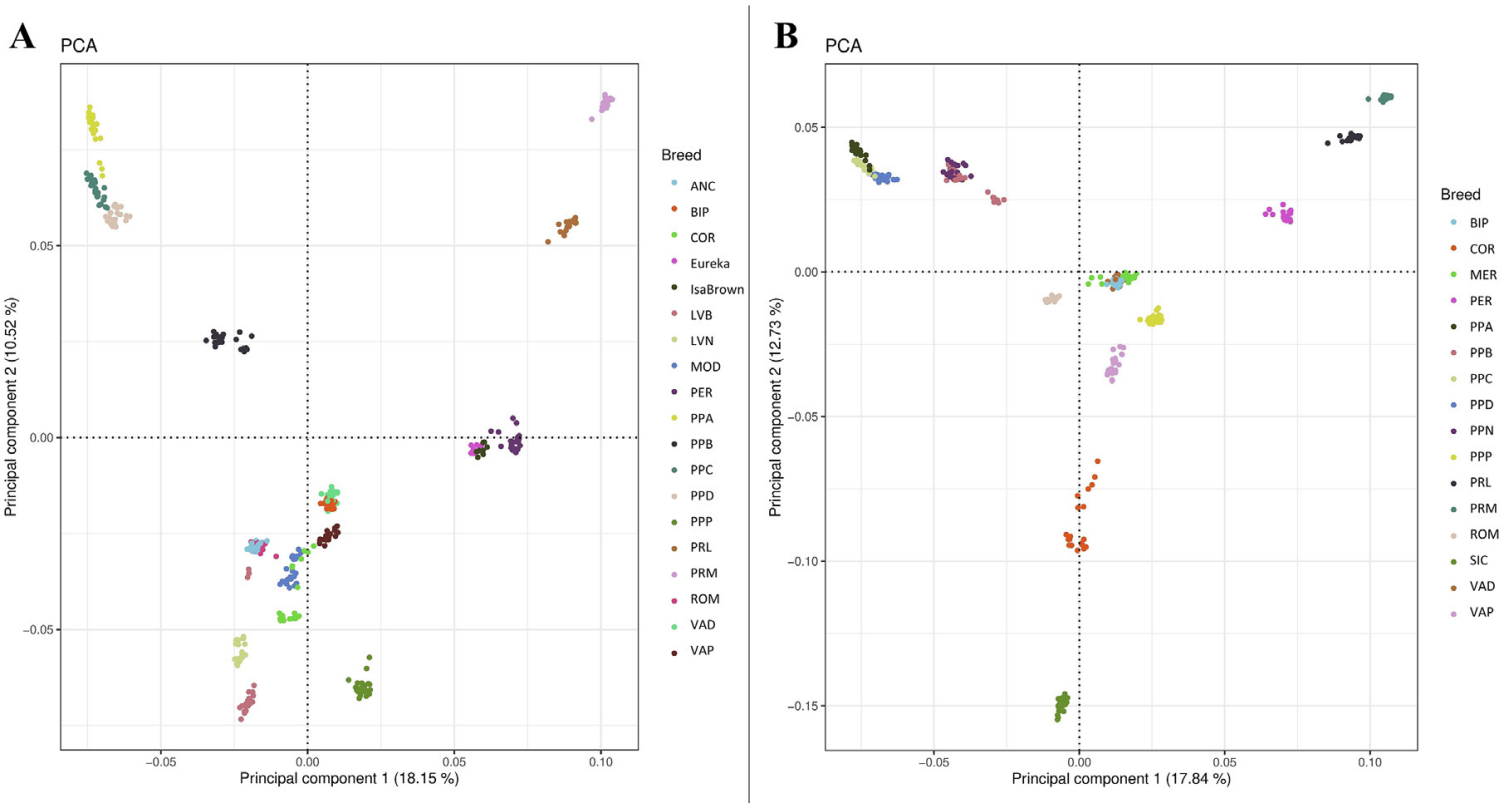
(A) Principal Component Analysis for the breeds used for eggshell phenotype analysis: Ancona (**ANC**), Bionda Piemontese (**BIP**), Eureka, IsaBrown, Livorno Bianca (**LVB**), Livorno Nera (**LVN**), Modenese (**MOD**), Ermellinata di Rovigo (**PER**), Padovana Argentata (**PPA**), Polverara Bianca (**PPB**), Padovana Camosciata (**PPC**), Padovana Dorata (**PPD**), Pepoi (**PPP**), Robusta Lionata (**PRL**), Robusta Maculata (**PRM**), Romagnola (**ROM**), Valdarnese (**VAD**), and Valplatani (**VAP**); (B) Principal Component Analysis for the breeds used for shank phenotype analysis: BIP, Cornuta di Caltanissetta (**COR**), Mericanel della Brianza (**MER**), PER, PPA, PPB, PPC, PPD, Polverara Nera (**PPN**), PRL, PRM, ROM, Siciliana (**SIC**), VAD, and VAP.

Following PLINK software v1.9 filtering, no animals were lost and SNPs which passed the quality control were 408,896 and 409,031 for eggshell and shank phenotypes, respectively. The PCA plots based on PC1 and PC2 depict the breeds clusterization with no outliers both in eggshell and shank groups (Figure 1). The PCA included breeds from both the case and control populations for the 2 phenotypes. The PCA indicated that the value of PC1 was approximately 18%, and values of PC2 were 10.52% and 12.73% for eggshell and shank, respectively.

In the GWAS analysis the relatedness matrices from PCA were taken into consideration for population structure correction. This approach allowed the linear mixed model used in GWAS to account for false positive signals by incorporating a random effect with a covariance matrix proportional to the relationship matrix (Mancin et al., 2021). The results of GWAS highlighted 92 significant SNPs mapped in 16 genes and 113 SNPs mapped in 27 genes in eggshell and shank analysis, respectively.

**Figure 2:**
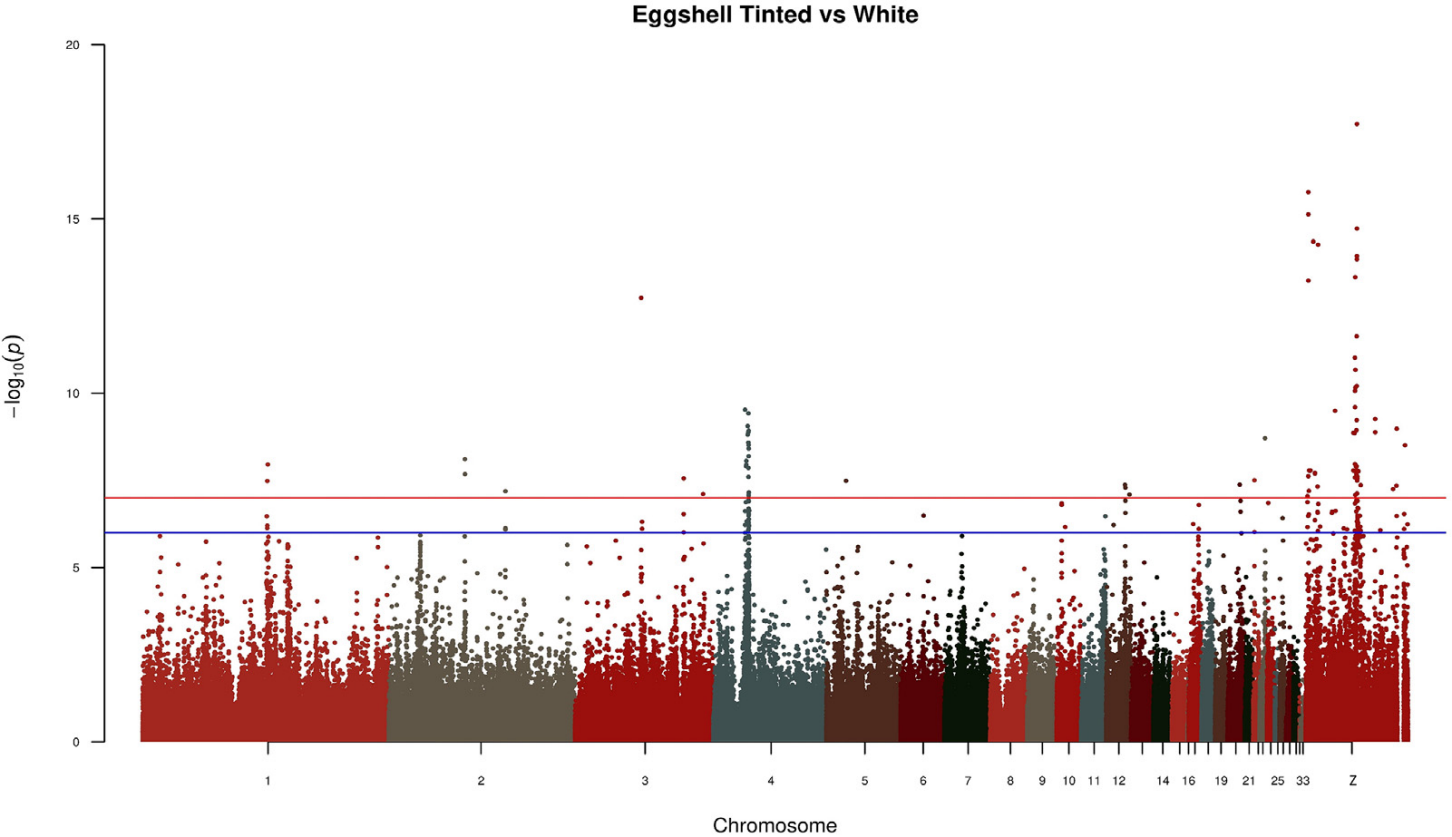
Manhattan plot of GWAS comparing case (tinted eggshell) and control population (white eggshell).

**Figure 3:**
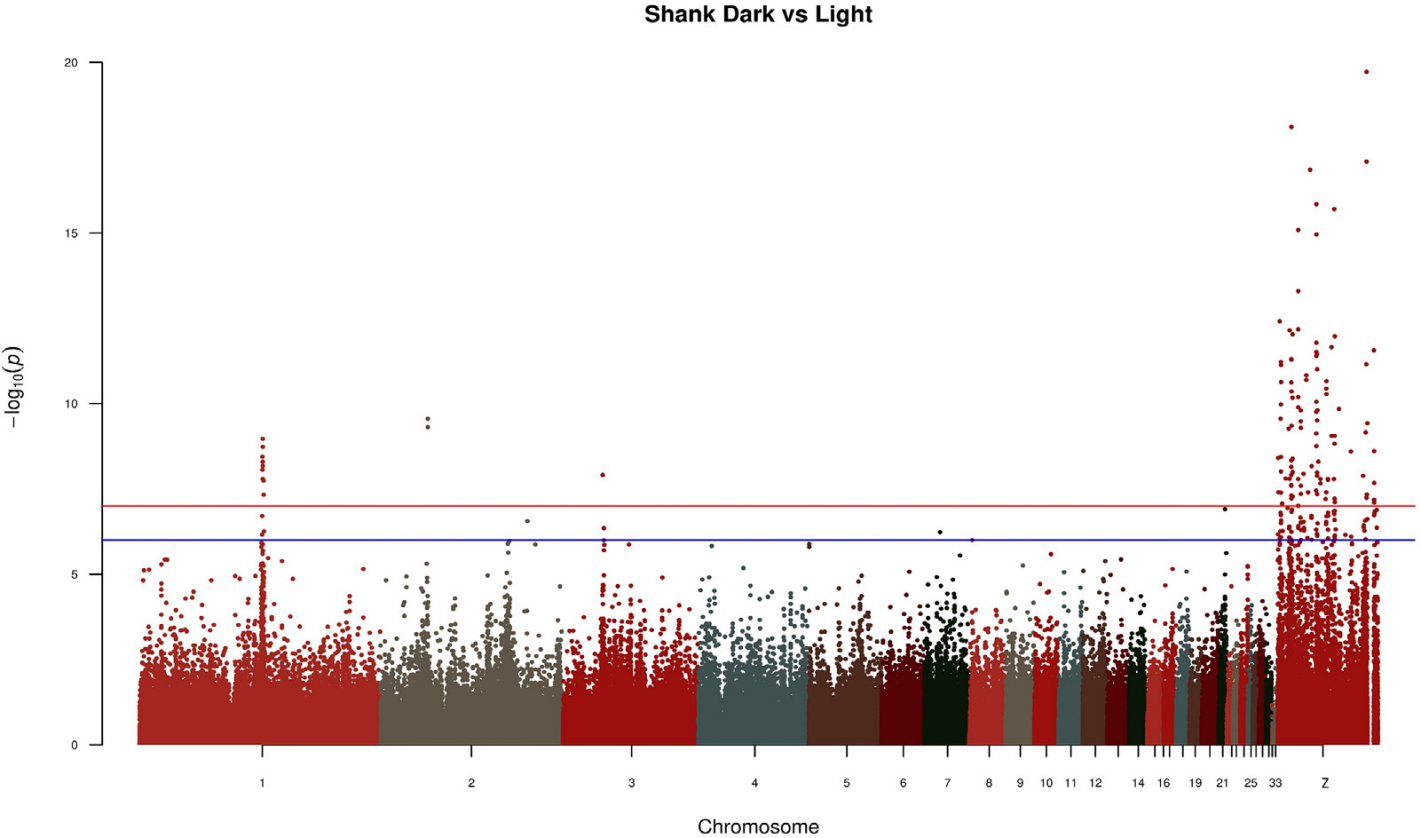
Manhattan plot of GWAS comparing case (dark shank) and control population (light shank).

**Figure 4:**
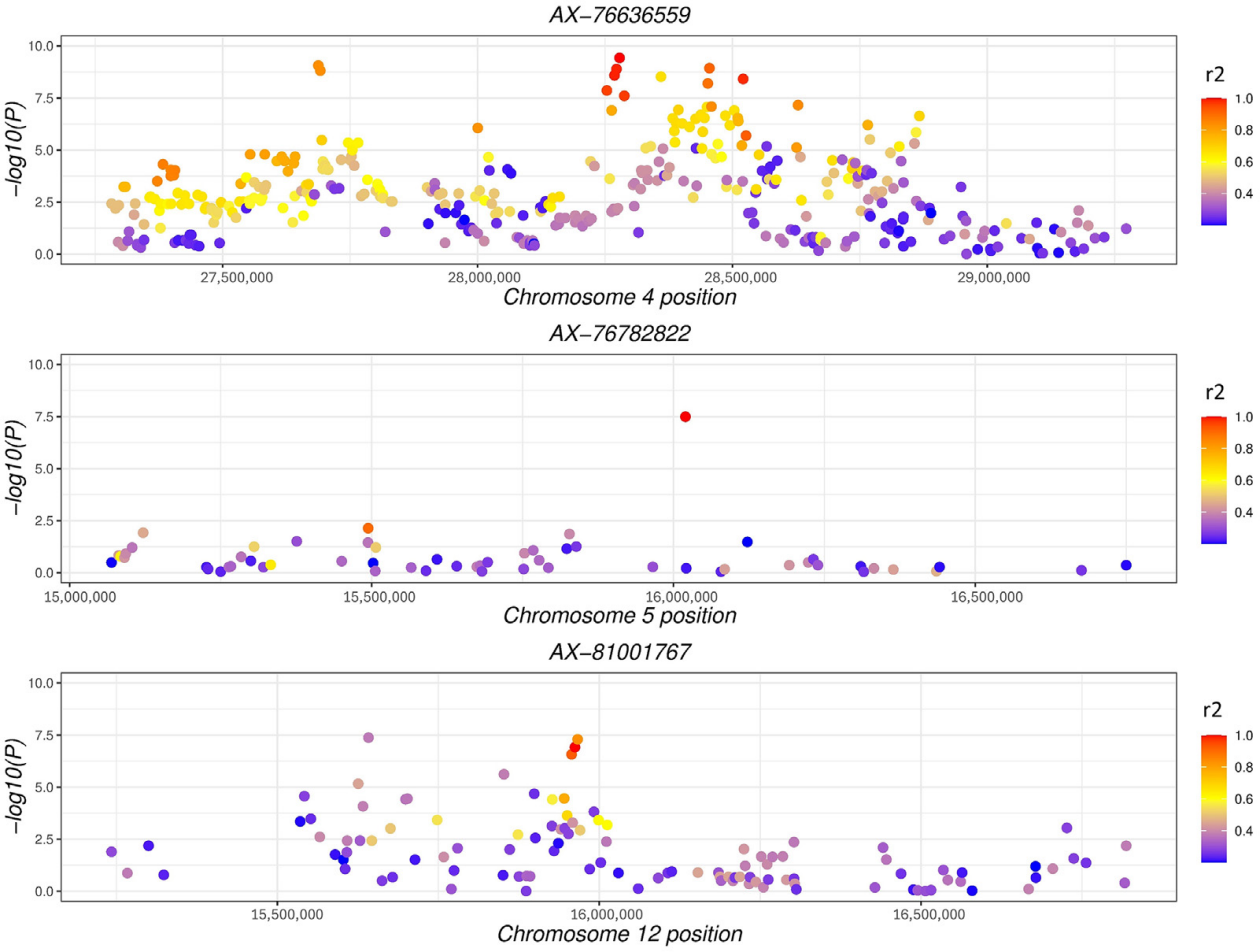
Linkage disequilibrium values of 3 selected SNPs in comparison to the region in which they are located for eggshell phenotype analysis. Y-axis represents the -log_10_ of *P*-value from GWAS eggshell analysis. The colors show the level of Linkage Disequilibrium (red = 1, blue = 0) of the SNPs in the regions regarding the selected SNP (AX-76636559 in chromosome 4, AX-76782822 in chromosome 5, AX-81001767 in chromosome 12).

**Figure 5:**
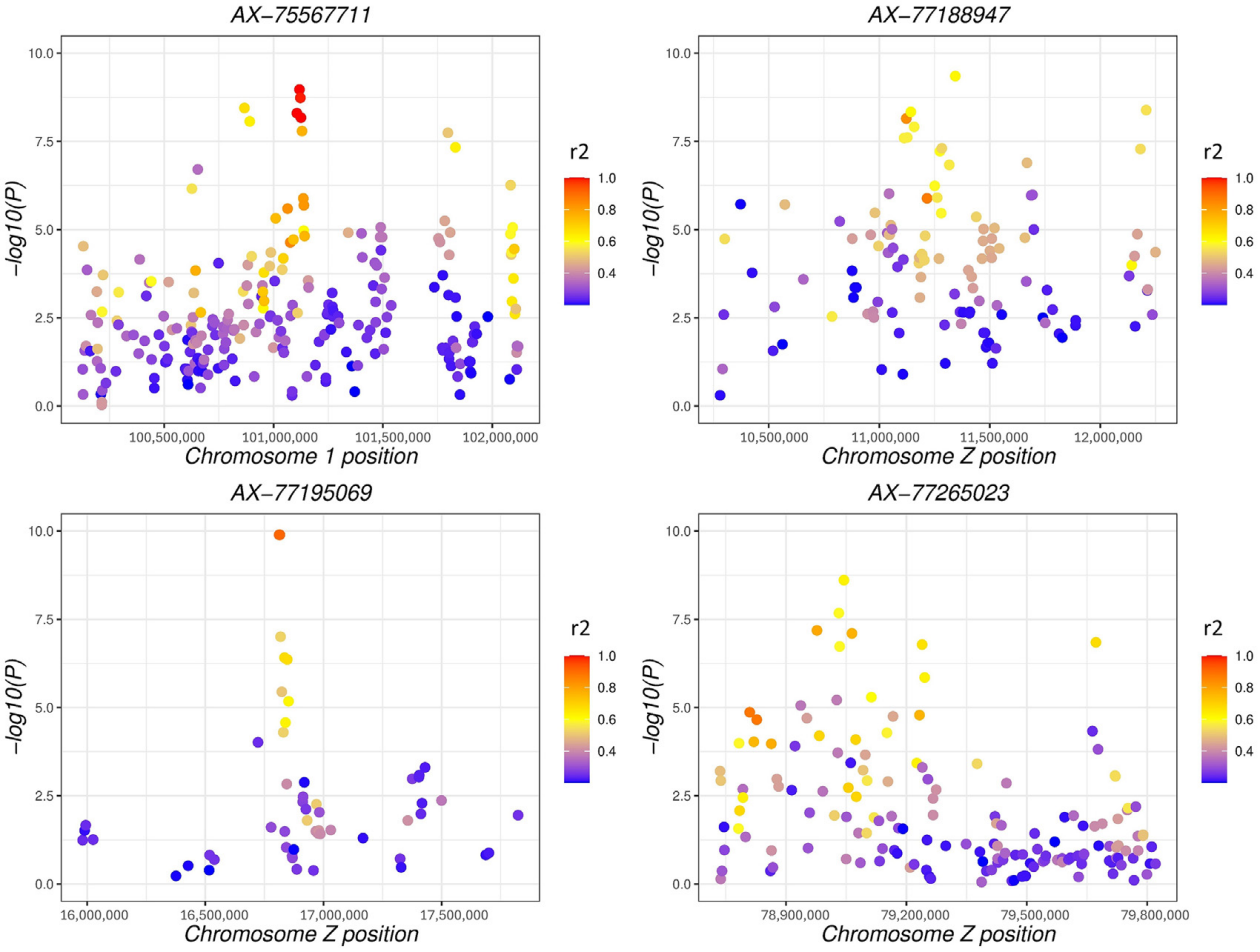
Linkage disequilibrium values of 3 selected SNPs in comparison to the region in which they are located for shank phenotype analysis. Y-axis represents the -log_10_ of *P*-value from GWAS shank analysis. The colors show the level of Linkage Disequilibrium (red = 1, blue = 0) of the SNPs in the regions regarding the selected SNP (AX-75567711 in chromosome 1, and AX-77188947, AX-77195069, and AX-77265023 in chromosome Z).

Figure 2 depicts the results from GWAS analysis in which the tinted eggshell breeds were used as case population and white eggshell breeds as control population. The lowest *P*-values were linked to SNPs mapped on chromosome Z. This applies also when comparing shank phenotypes, namely dark (case population) vs. light (control population; Figure 3). Despite the GWAS analysis showed significant SNPs mapped on GGA Z, the annotations did not highlight regions in common. Figure 2 and Supplementary Material 1 report a specific genomic region (Z:39637983-40955369) encompassing the genes *FRMD3, GKAP1, NTRK2, AGTPBP1, ISCA1, NAA35, ZCCHC6, GAS1*, and *DAPK1*. As regards shank phenotype, significant SNPs in Z chromosome associated to regions of interest were observed (Figure 3 and Supplementary Material 1). Table 1 shows the genes annotated in the genomic regions of GWAS which resulted significant and closely related to both phenotypes. The genes *SLC1A3* and *RANBP3L* mapped on Z:11112490-12254700, which is a region extremely close to the gene *SLC45A2*. The region Z:16807993-16830690 features the presence of the *PLPP1* and *SLC38A9* genes. Furthermore, other regions of interest were identified, in which *ERCC8, BNC2,* and *CAMK4* genes were annotated (Table 1). Three regions strictly related to shank pigmentation were found in the chromosome Z; the first linked to *CDKN2A* and *CDKN2B*, the second associated to *MTAP* and *FEM1C*, and the third containing the genes *TIM36* and *GRAMD3*. For both the phenotypes under investigation, highly significant *P*-values were identified for SNPs mapped on the autosomes. For instance, significant SNPs mapped on GGA 1, GGA 2, and GGA 3 out of the shank phenotype analysis. Moreover, the GGA 1, GGA 2, GGA 3, GGA 4, GGA 5, GGA 12, GGA 20, GGA 22, and GGA 23 owned SNPs with the lowest *P*-values in relation to eggshell phenotype (Figures 2 and 3). Supplementary Material 1 reports the genes that have been annotated in the aforementioned chromosomes, with those most related to the given phenotype presented in Table 1. For the SNPs of interest, the *F*_ST_ and LD were calculated. Figure 4, Figure 5 depict LD values of selected SNPs extrapolated from eggshell and shank analysis, respectively. Regarding the eggshell phenotype, 3 SNPs were chosen on the basis of their location inside or close to genes of interest for both *F*_ST_ and LD values: AX-76636559 (*P*-value < 3.77^−10^), AX-76782822 (*P*-value < 3.24^−08^), and AX-81001767 (*P*-value < 5.06^−08^) (Supplementary Material 1). The mentioned SNPs were related to *PCDH18, SLC25A22*, and *MITF* genes, respectively (Table 1). The same SNPs were selected to calculate the *F*_ST_ and the results are presented in Supplementary Figure 1. Regarding shank phenotype, 4 SNPs were taken into consideration and the LD values compared to their genomic regions were calculated (Figure 5). The SNP AX-75567711 was the only mapped on autosome among the 4 reported here and was located close to *CHDOL* and *TMPRSS15* genes. Other 3 SNPs were all located on GGA Z close to *SLC1A3, SLC38A9*, and *MTAP* genes, respectively (Table 1). AX-77265023, the last SNP reported in Figure 5 and mapped in an intronic section of gene *MTAP*, showed low *P*-value (2.75^−12^) and high frequency in the dark population of shank phenotype (0.405) (Supplementary Material 1). The *F*_ST_ values are presented in Supplementary Figure 2 regarding the same 4 SNPs analysed in in Figure 5.

**Table 1:** Chromosome and position, gene ID, description of gene function, and reference of shank and eggshell phenotypes.

**SHANK**			
*Chromosome and position*	*Gene ID*	*Description*	*Reference*
1:100867039-101831504	*CHODL, TMPRSS15, NCAM2*	Genomic region related to horn pigmentation in cattle	Zsolnai et al. (2021)
2:38800153-38800475	*EOMES, CMC1, AZI2, RBMS3*	Expression enhanced in embryos of chicken with double comb	Dorshorst et al. (2015)
Z:11112490-12254700	*SLC1A3, RANBP3L, SLC45A2*	SLC family is implicated in pigmentation in white tiger, and SLC1A3 is upregulated in development of stem cells in hair follicles	Xu et al. (2013)
Z: 18925613-18969905	*ERCC8*	Groningen White Headed cattle	Gonzalez-Prendes et al. (2022)
Z: 31545096-32699330	*NFIB, ZDHHC21, CER1, PSIP1, BNC2, TYRP1*	BNC2 strongly associated in human skin pigmentation	Jacobs et al. (2013)
Z: 78827158-79172113	*CDKN2A, CDKN2B*	Play role in the barring phenotype of the chicken by altering the melanocyte cell cycle	Dorshorst and Ashwell (2009)
Z: 78827158-79172113	*MTAP, FEM1C*	Associated with skin and shank pigmentation in chicken	Cha et al. (2023)
Z: 78827158-79213873	*TRIM36, GRAMD3*	Associated with skin and shank pigmentation in chicken	Li et al. (2014)
**EGGSHELL**			
2:61184687-61271239	*JARID2*	Involved in regulation of gene expression during embryonic development	Whiteley et al. (2021)
4:26751167-30211214	*PCDH18*	*PCDH18* very close to *SLC7A11* responsible for coloration in mammals (of skin) and in chicken plumage and skin	Chen et al. (2019)
5:15963249-15987149	*PNPLA2, SLC25A22*	Related with production of carotenoids	Ahi et al. (2020)
12:15839118-16014277	*MITF, FAM19A4, ARL6IP5, UBA3*	Linked to pigmentation in cattle and other species, in duck associated with pigmentation of beak	Gonzalez-Prendes et al. (2022)
Z:10028822-10307280	*NPR3, TARS, ADAMTS12, SLC45A2*	*SLC45A2* is one of the most important gene in coloration	Dorshorst and Ashwell (2009)

## DISCUSSION

### Loci Associated With Shank Pigmentation in Autosomes

A new locus never described before in chicken and related to pigmentation is the one mapped on GGA 1 around 100 Mb. Here, 3 genes are located, namely Chondrolectin (***CHODL***)*,* Transmembrane Serine Protease 15 (***TMPRSS15***), and Neural Cell Adhesion Molecule 2 (***NCAM2***)*,* which are involved in horn pigmentation in cattle (Zsolnai et al., 2021). In detail, *NCAM2* is implicated in the chondrogenesis in chicken embryo (Fang and Hall, 1995), hence it could have a low *P*-value due to its involvement in general shank phenotype (like in cattle horn phenotype, according to Zsolnai et al., 2021), but not specifically in pigmentation of the shank. The role of *NCAM2* is uncertain but for the first time it seems to be related to shank pigmentation. The region in GGA 2 (Table 1) which in particular includes the gene Eomesodermin (***EOMES***) is related to the V-combe and Buttercup-comb phenotypes, both reported in the Sicilian breed COR (Dorshorst et al., 2015). Dorshorst et al. (2015) characterized the high expression of the *EOMES* gene in the ectoderm as the trigger signal of duplication event of the comb region, leading to the majority of the comb duplication phenotype. Indeed, the COR and SIC breeds are part of the dark shank phenotype group used as case study population, but since they are both the only breeds in the dataset with this type of phenotype, the signal in GWAS was related to the comb.

### Loci Associated With Shank Pigmentation in Z Chromosome

The remaining GWAS signals for dark shank are located in GGA Z. Of particular interest for the shank phenotype is the locus located at 31 Mb in GGA Z. Specifically, a SNP exhibited a remarkably low *P*-value (<1.44^−16^) and another SNP belonging to the same genomic region was mapped within an intronic region of the Nuclear Factor I B gene (***NFIB***)*.* As reported by several studies, this position in GGA Z is related to dark skin and shank due to the presence of gene *TYRP1* close to *NFIB* gene (500 Kb) (Li et al., 2019; Khumpeerawat et al., 2021). The *TYRP1* is a catalase enzyme which is involved in the production of eumelanin. A *TYRP1* mutation in a zinc-binding region can produce melanocytes that have more melanosomes, although they are granular and irregular in structure (Andersson, 2020). Another interesting gene, the Excision Repair 8 (***ERCC8***) has been previously linked to a human genetic disorder characterized by differentiated skin pigmentation and increased freckling (Table 1; Nardo et al., 2009). In Groningen White Headed cattle, the *ERCC8* gene is implicated in the pathway of skin pigmentation bringing to the peculiar phenotype of the breed characterized by black or red body and white head. The role of *ERCC8* is important for UV protection and this could impact the final colour of shank in chicken (Gonzalez-Prendes et al., 2022). A real interesting region is Z:31376546-32699330 in which the gene Basonuclin 2 (***BNC2***) has been found; this gene is strongly associated with human different saturation skin pigmentations (Jacobs et al., 2013). The literature reports that *BNC2* is connected to the darkness of the skin. For instance, Visser et al. (2014) observed that higher expression of *BNC2* is associated with dark skin. Consistently, it is reported to be implicated in freckles and facial pigmented spots during aging, and also in keratinocyte carcinoma in human (Jacobs et al., 2015; Kim et al., 2022). The *BNC2* has not been reported before in chicken and especially linked to the dark pigmentation of the shank. In the present study the association is underlined by the SNP AX-77212407 which mapped on the intronic region of the gene; this SNP showed low *P*-value and MAF of 0.444, meaning that the majority of the case population (dark shank) owned the minor allele. Furthermore, this QTL is mapped 500 Kb upstream to *TYRP1*, a pivotal gene in pigmentation of skin and plumage in chicken. Indeed, a *TYRP1* missense mutation creates chocolate plumage in chickens and changes melanosome structure (Li et al., 2019). Moreover, *TYRP1* has been found significantly more expressed in the black chicken meat when compared to the white one in Tengchong Snow chickens breed (Zi et al., 2023), and it was higher expressed in black skin birds compared to white birds among Thailand chicken breeds (Khumpeerawat et al., 2021). The last region of interest is located in the distal end of chromosome Z (Table 1) from 78,846,780 to 79,213,873 bp. This chromosome area contains genes of interest such as Cyclin Dependent Kinase Inhibitor 2A and 2B (***CDKN2A*** and ***CDKN2B***), Methylthioadenosine Phosphorylase (***MTAP***), Fem-1 Homolog C (***FEM1C***), Tripartite Motif Containing 36 (***TRIM36***), and GRAM Domain Containing (***GRAMD3***) which have been already reported to be implicated in pigmentation and some of them specifically linked to dark shank pigmentation. The *CDKN2A* and *CDKN2B* are tumor-suppressor genes which encodes the Alternate Reading Frame (**ARF**) protein (Hellstrom et al., 2010). The sex-linked barring causes premature death of the melanocytes, and this process is ascribable to mutation in *CDKN2A* promotor and/or intron, meaning that this locus is correlated with melanocyte biology function (Hellstrom et al., 2010). The ARF reduces the E3 ubiquitin-protein ligase *MDM2* activity, leading to the degradation of the *p53* tumor suppressor-transcription factor. This could explain the reason why these mutations impair pigmentation. Thus, the upregulation of ARF by the *B0* mutation(s) blocks *MDM2*, and thereby upregulates *p53*, which results in a loss of pigment cells (Andersson, 2020). In line with Andersson et al. (2020), *CDKN2A* and *CDKN2B* are reported to influence the barring phenotype in chicken as well as *MTAP* and *TRIM36* genes (Dorshorst and Ashwell, 2009). One candidate gene for shank pigmentation in the present analysis is the *MTAP* because 2 SNPs were found strongly significant, with MAF of 0.405, meaning a strong segregation of minor allele in dark population. The *MTAP* gene encodes methyl-thioadenosine phosphorylase, acting in the methionine salvage pathway (Kirovski et al., 2011). The *MTAP* gene has been already identified in chicken as main responsible of skin pigmentation (Cha et al., 2023), hyperpigmentation in the Silkie chicken (Tian et al., 2014), and pigmentation of Tibetan chicken shank (Li et al., 2014). In particular, Li et al. (2014) used the same DNA chip of the present study and found the QTL of *MTAP* and *CDKN2A/B* using the same GWAS approach. The authors analyzed only animals of the Tibetan chicken breed, with 19 hens exhibiting dermal pigmentation shank and 21 hens exhibiting yellow shank.

Other 2 genomic regions were discovered in the present analysis, both belonging to solute carrier family (**SLC**) and mapped on chromosome Z. The first one is represented by *SLC1A3*, a gene mapped 700 Kb far from another member of SLC family, the *SLC45A2* gene. The *SLC1A3* gene is a glutamate transporter which mediates inter-niche stem cell activation during skin growth (Reichenbach et al., 2018), whereas the *SLC45A2* gene is one of the most responsible for skin and dermal pigmentation, and its mutations result in the decrease or complete inhibition of the synthesis of one or both types of melanin in domestic chicken, quails, tiger, and other species (Gunnarsson et al., 2007; Soejima and Koda, 2007; Xu et al., 2013). Finally, the last region in shank phenotype is related to GGA Z in 16 Mb position owning the Phospholipid Phosphatase 1 (***PLPP1***) and *SLC38A9* genes.

### Eggshell

For eggshell color, less regions were identified compared to shank color. The first was in GGA 2 and it is represented by the gene Jumonji and AT-rich interaction domain containing 2 (***JARID2***). The *JARID2* gene is implicated in DNA binding, nuclear localization, and transcriptional repression (Pasini et al., 2010). Moreover, it has been identified in White Leghorn chickens as a divergent gene when compared to Dongxiang Blue-Shelled chicken (Zhao et al., 2018).

Interesting QTLs in GGA 4 and GGA 12 included the cystine glutamate transporter *(**SLC7A11**)* and the Melanocyte Inducing Transcription Factor *(****MITF****)*, respectively, both involved in pigmentation (Table 1). The *SLC7A11* is a membrane protein that transports cysteine from the extracellular space to the cytosol of melanocytes. The melanocytes obtain the sulfhydryl group required for pheomelanin synthesis from cysteine. The *SLC7A11* expression correlates with *MITF* expression, because *MITF* induces *SLC7A11* transcription. The *MITF* and *SLC7A11* genes are implicated in skin pigmentation in different species, such as quail (Minvielle et al., 2010), rabbit (Chen et al., 2019), and tawny owl (Emaresi et al., 2013). Also, *MITF* promoter polymorphisms affected chicken skin colour and transcriptional activity in Black-boned chickens (Wang et al., 2017).

The pathways, related genes, and enzymes involved in the pigmentation of chicken eggshell are partially known: brownness eggshell levels are in charge of the abundance of an immediate precursor of hem, the Protoporphyrin IX (Bi et al., 2018), whereas the blue eggshell is mainly up to deposition of biliverdin, a byproduct of the breakdown of hemoglobin (Hargitai et al., 2017). The pathway involved in the production and deposition of these pigments in eggshell is partially known. The main genes involved in the process of brownness eggs are 5′-Aminolevulinate Synthase 1 (***ALAS1***), ATP Binding Cassette Subfamily G Member 2 (***ABCG2***), FLVCR Choline And Heme Transporter 1 (***FLVCR1***), *SLC25A38,* and genes belonging to *COX* family (Yang et al., 2022). *ALAS1* is the gene thought to have pivotal role in the production of the pigment Protoporphyrin IX, whereas the others mentioned genes are transporter, as they influence the deposition of pigments on the eggshell. In addition to the *SLC7A11* gene, other transporters were identified in the current study, such as *SLC25A22* on GGA 5 and *SLC45A2* on GGA Z. While the *SLC45A2* gene has been already discussed in relation to the shank phenotype, *SLC25A22* is a significant mitochondrial glutamate transporter (Reid et al., 2017) although it has not been previously linked to any pigmentation phenotype in any species. During the final stage of egg formation, the egg enters the shell gland (the uterine part of the oviduct), and the pigments are secreted into the uterine fluid and gradually laid down in the eggshell (Wang et al., 2017). As a result, the carrier protein plays a role in eggshell pigmentation, which may explain why we discovered the low *P*-value signals around genes coding for cell transporters.

In conclusion, this comprehensive analysis shed light on the genetic architecture underpinning shank and eggshell colour in chickens. The results offer novel insights into the genes and pathways involved in chicken pigmentation, suggesting *MTAP* and *TYRP1* as candidate genes for dark shank phenotype. Moreover, genes included in solute carrier family seem to have a pivotal role in deposition of pigments in eggshell. Although further research is needed to explore the functional significance of the identified genes and their role in avian pigmentation regulation, the present paper reported important genes related to the phenotypic features with a pivotal role in the maintenance of breed standards.

## Appendix Supplementary materials

Supplementary Material 1. Annotation for variants with *P*-value < 5 × 10^−8^ from GWAS analysis. The first 2 sheets report annotation for eggshell phenotype analysis and the last 2 sheets for shank phenotype analysis. Sheet 1 and sheet 3 report the annotation for each SNP with a significant *P*-value identified in the GWAS analysis for eggshell and shank, respectively, performed by SnpEff software. Columns of sheet 1 and 3 are: chromosome (*Chromosome*); position of the variant in the chromosome (*Position*); name of the variant as in Affymetrix chip array (*Id*); ID of the variant used in Ensembl (*Existing Variation*); number of missing genotypes in the dataset for the SNP (*n miss*); variant allele (*allele1*); common allele (*allele0*); allele frequency of allele1 (*af*); symbol of the gene if the SNP is mapped inside a gene (*Symbol*); Ensembl nomenclature for gene in which the SNP is located (*Gene*); biological type of SNP (*Biotype*); and p-wald of the SNP from the GWAS (*p-wald*). Sheet 2 and sheet 4 report the annotation performed by GALLO software of the SNPs with *P*-value < 5 × 10^−8^ using a range of 0.1 Mb around the selected SNP for eggshell and shank, respectively. Columns of sheet 2 and sheet 4 include: chromosome (*Chromosome*); SNP position (*Position*); start position of the annotated gene (*Start_pos*); end position of the annotated gene (*End_pos*); width of the annotated gene (*Width*); Ensembl ID of the annotated gene (*Gene_id*); common name of the annotated gene (*Gene_name*); and biological type of the annotated gene (*Gene_biotype*). Image, application 1

Supplementary Figure 1. *F*_ST_ values referred to position around the same selected SNPs for Linkage Disequilibrium analysis in Figure 4. Y-axis represents the *F*_ST_ value (from 0 to 1) of each SNP located in the region reported in x-axis. The analysis is referred to eggshell phenotype. Image, image 2

Supplementary Figure 2. *F*_ST_ values referred to position around the same selected SNPs for Linkage Disequilibrium analysis in Figure 5. Y-axis represents the *F*_ST_ value (from 0 to 1) of each SNP located in the region reported in x-axis. The analysis is referred to shank phenotype. Image, image 3

Supplementary Figure 3. Quantile–quantile plots of P-values after Bonferroni correction in both GWAS (Figures A and B represent eggshell and shank analysis, respectively). The x-axis indicates the expected −log_10_ (P values) and the y-axis the observed −log_10_ (P-values). The figure reports the λ values for both the analyses. Image, image 4

Supplementary Table 1. Italian local chicken breeds grouped according to shank pigmentation. Image, application 5

## Footnotes

Supplementary material associated with this article can be found, in the online version, at doi:10.1016/j.psj.2024.103677.
