# Supplementary figures and images for: Genomic insights into shank and eggshell color in Italian local chickens
Source: Poult Sci. 2024 Mar 21;103(6):103677. doi: 10.1016/j.psj.2024.103677 (PMC11004871; doi:10.1016/j.psj.2024.103677)

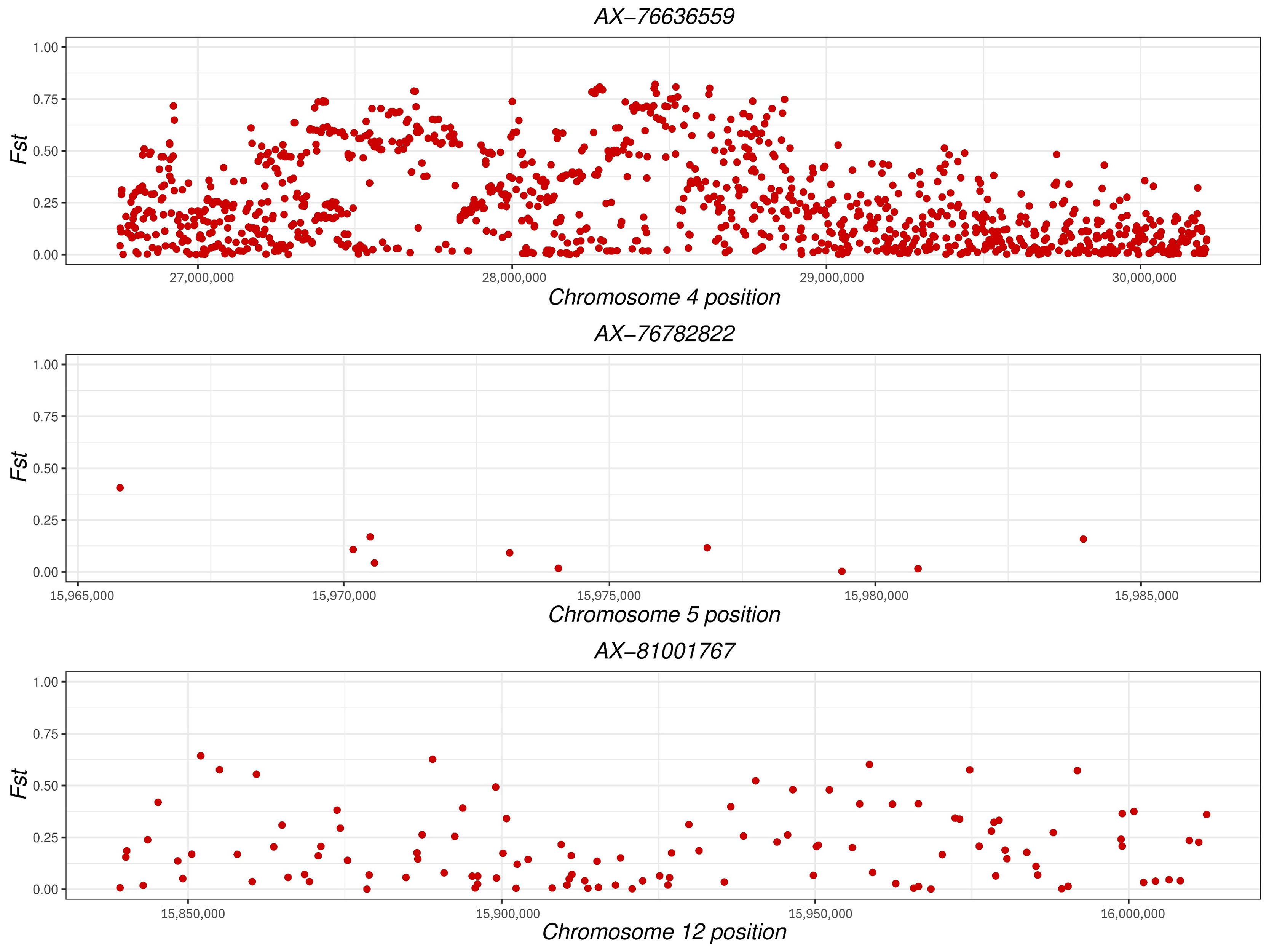

Supplement: Supplementary file 2 — Supplementary Figure 1. FST values referred to position around the same selected SNPs for Linkage Disequilibrium analysis in Figure 4. Y-axis represents the FST value (from 0 to 1) of each SNP located in the region reported in x-axis. The analysis is referred to eggshell phenotype. [file mmc2.jpg]

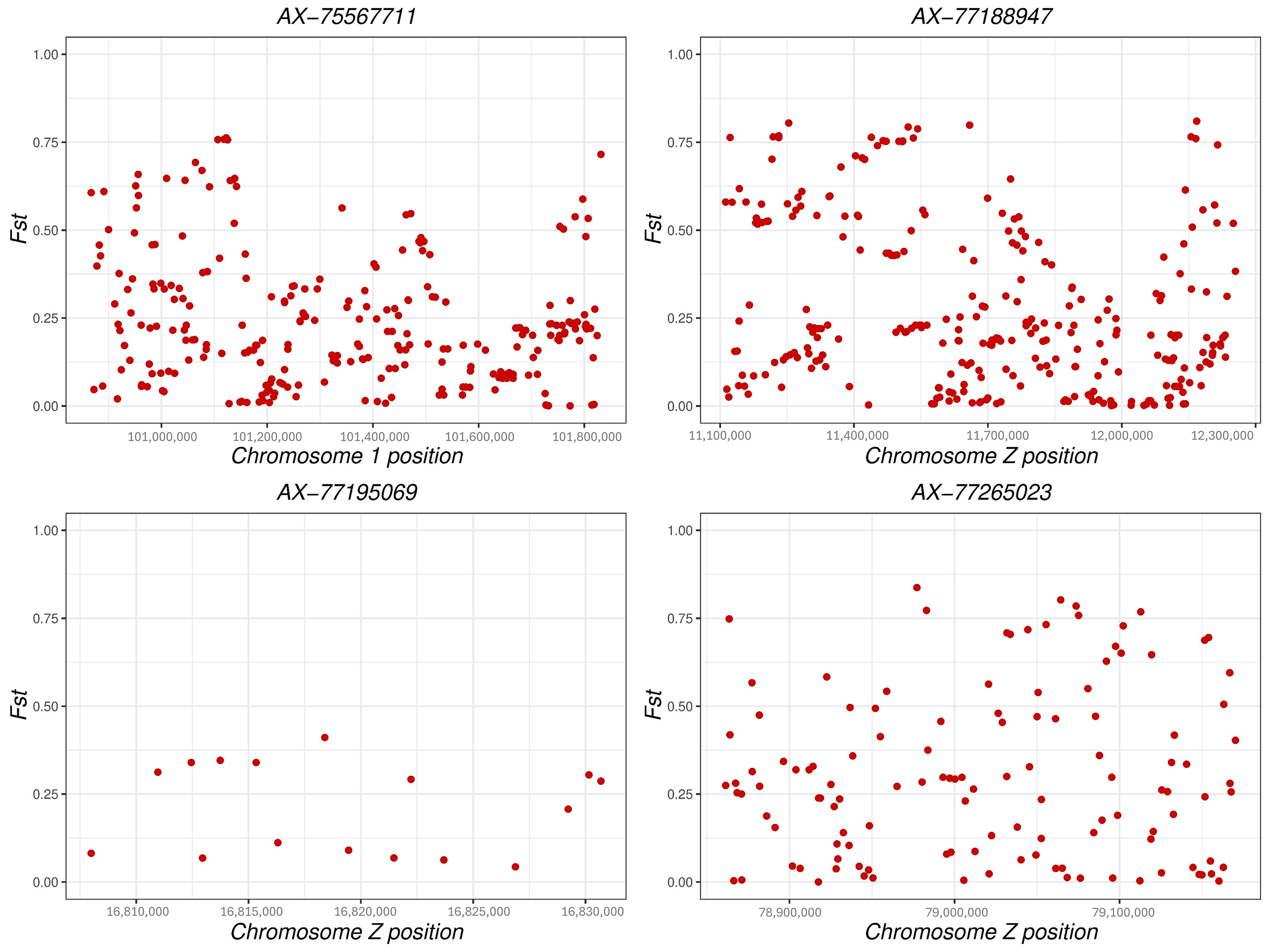

Supplement: Supplementary file 3 — Supplementary Figure 2. FST values referred to position around the same selected SNPs for Linkage Disequilibrium analysis in Figure 5. Y-axis represents the FST value (from 0 to 1) of each SNP located in the region reported in x-axis. The analysis is referred to shank phenotype. [file mmc3.jpg]

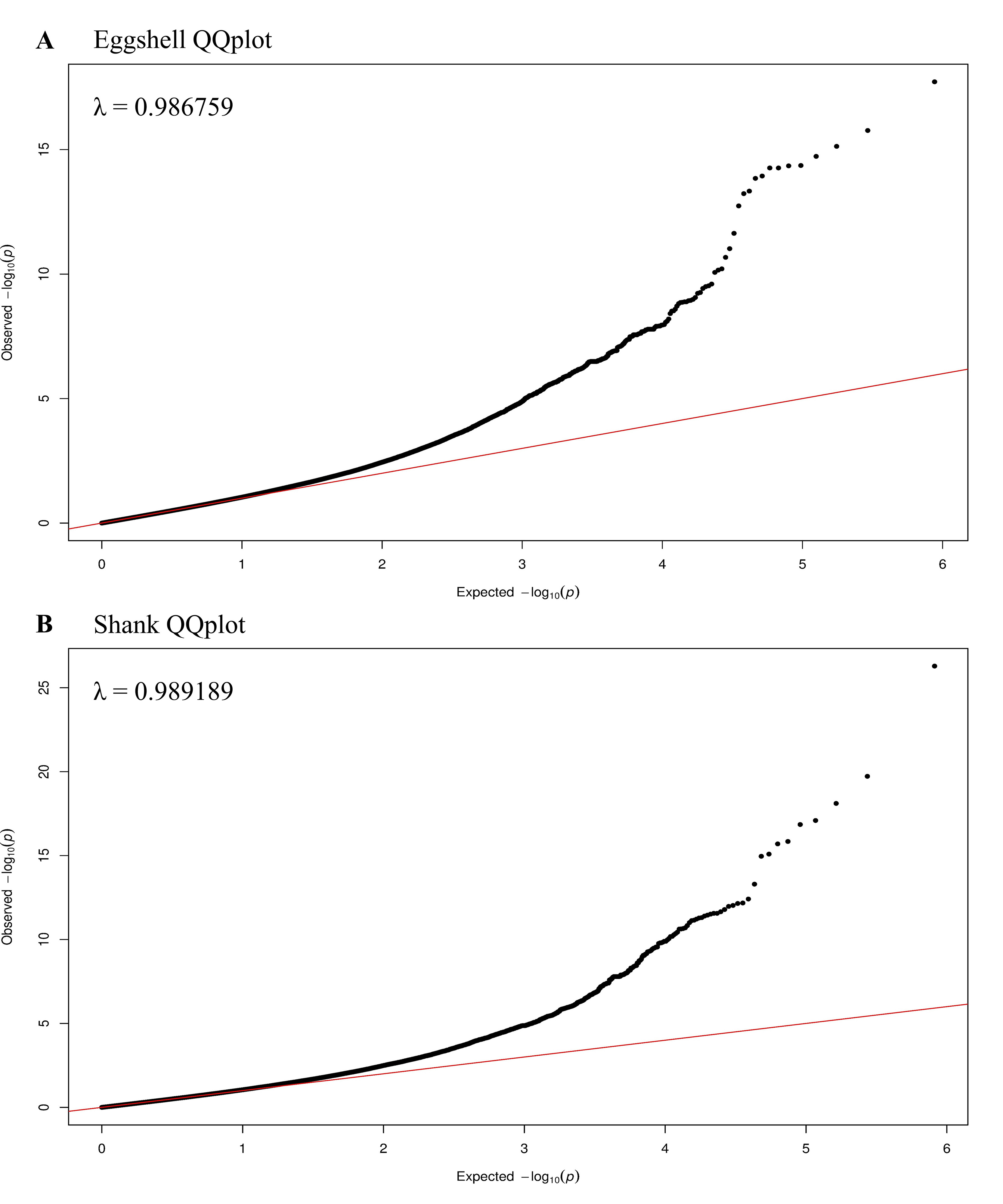

Supplement: Supplementary file 4 — Supplementary Figure 3. Quantile–quantile plots of P-values after Bonferroni correction in both GWAS (Figures A and B represent eggshell and shank analysis, respectively). The x-axis indicates the expected −log10 (P values) and the y-axis the observed −log10 (P-values). The figure reports the λ values for both the analyses. [file mmc4.jpg]
