# Supplementary material for: Genomic insights into shank and eggshell color in Italian local chickens
Source: Poult Sci. 2024 Mar 21;103(6):103677. doi: 10.1016/j.psj.2024.103677 (PMC11004871; doi:10.1016/j.psj.2024.103677)
Supplement: Supplementary file 5 — Supplementary Table 1. Italian local chicken breeds grouped according to shank pigmentation. [file mmc5.docx]

| **DARK** | | **LIGHT** | |
| --- | --- | --- | --- |
| 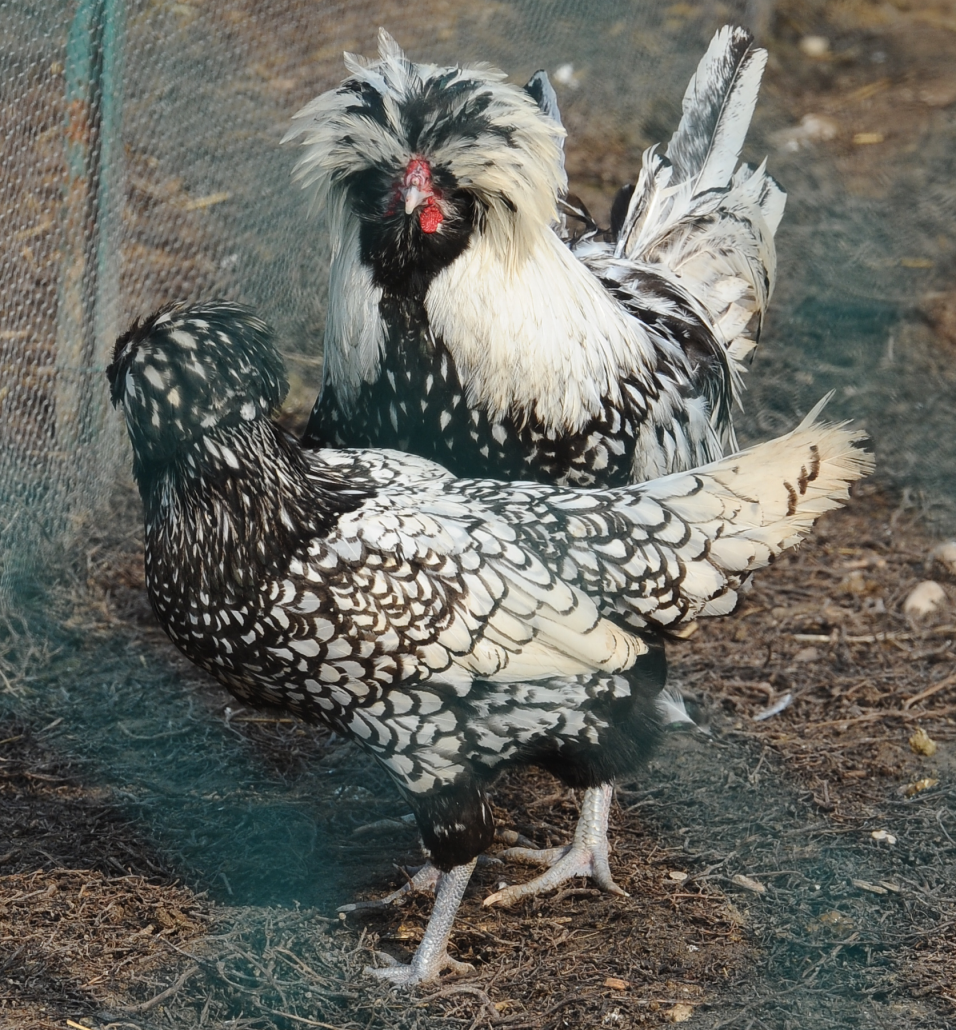 | Padovana Argentata  (PPA) | Bionda Piemontese  (BIP) | 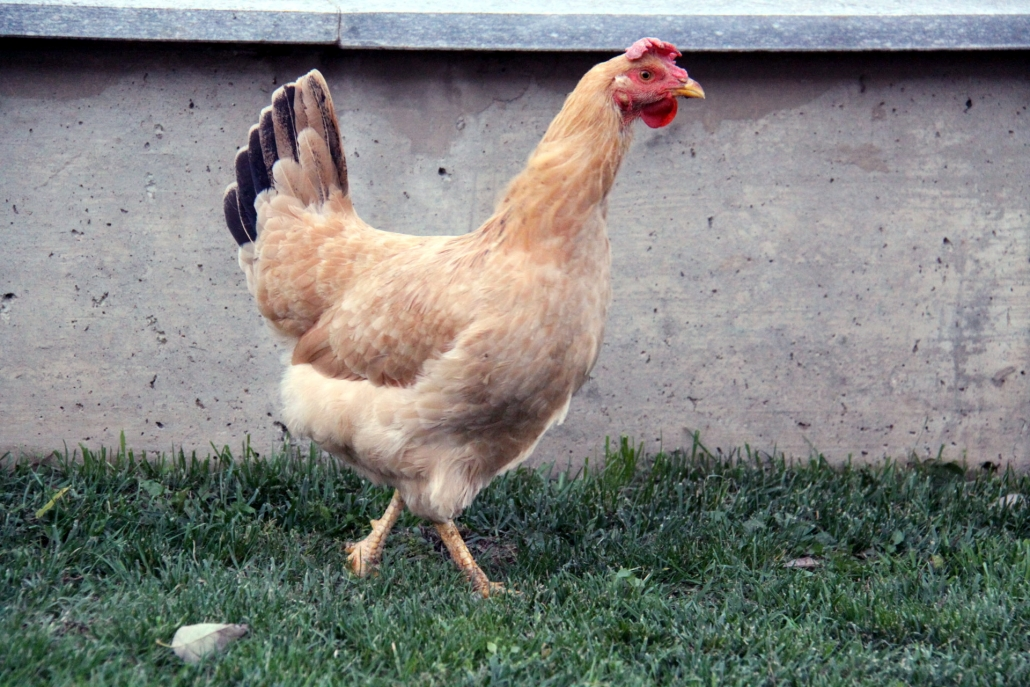 |
| 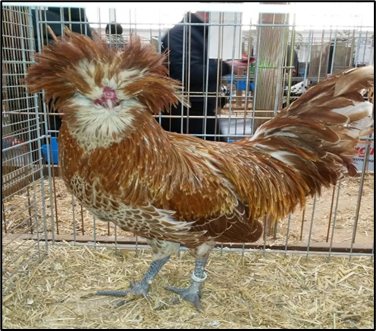 | Padovana Camosciata  (PPC) | Ermellinata di Rovigo  (PER) | 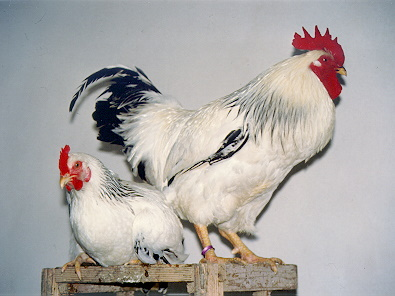 |
| 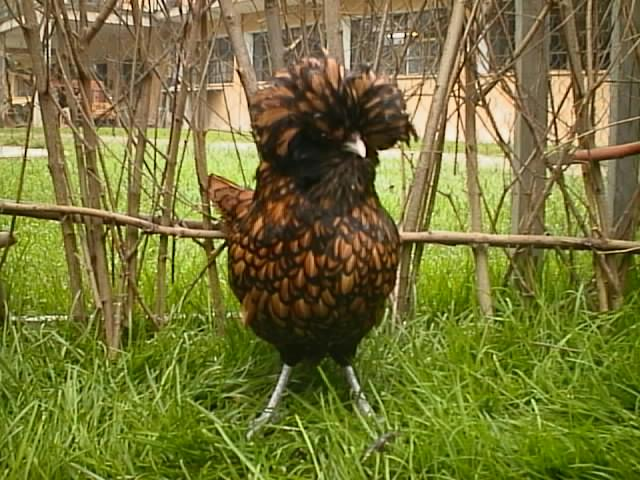 | Padovana Dorata  (PPD) | Mericanel della Brianza  (MER) | 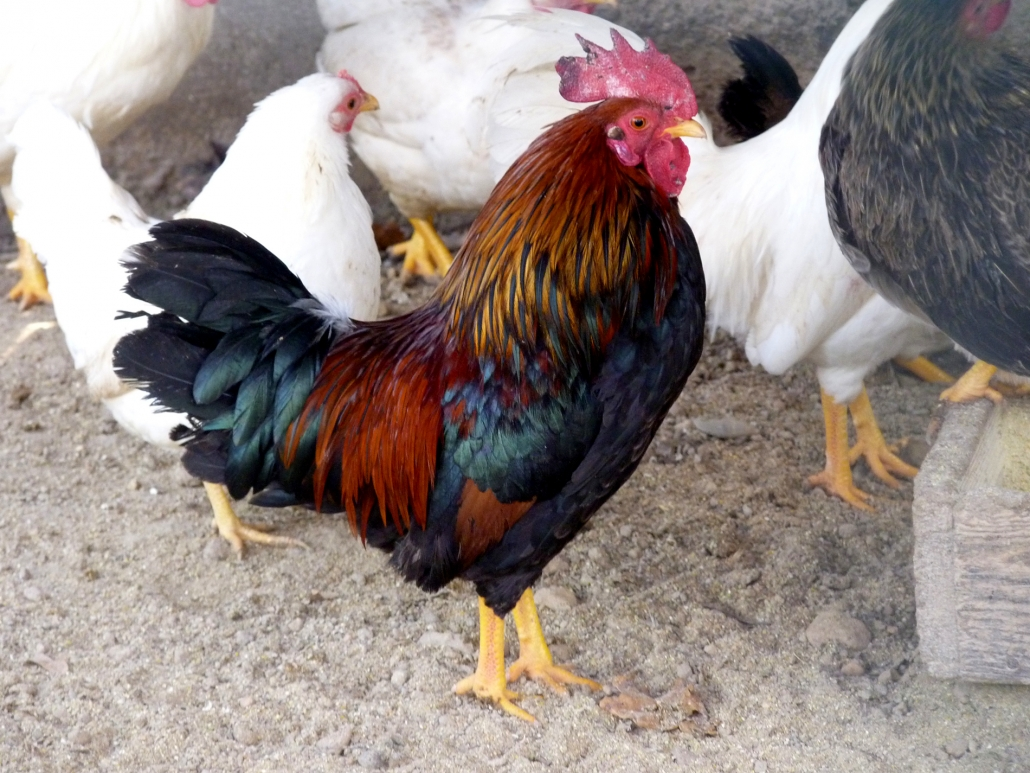 |
| 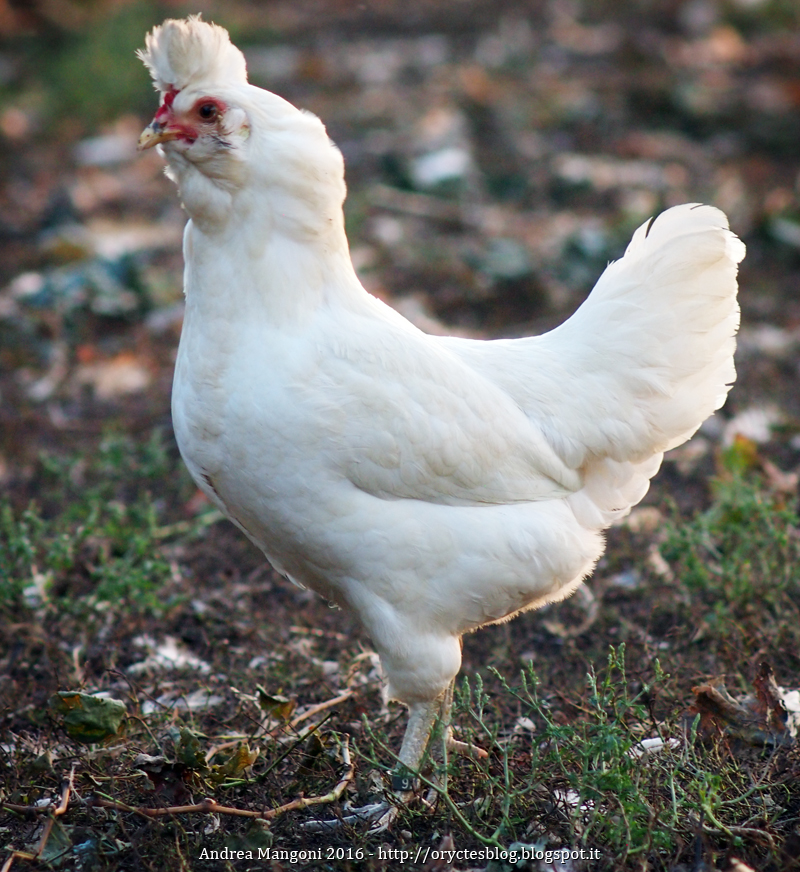 | Polverara Bianca  (PPB) | Pepoi  (PPP) | 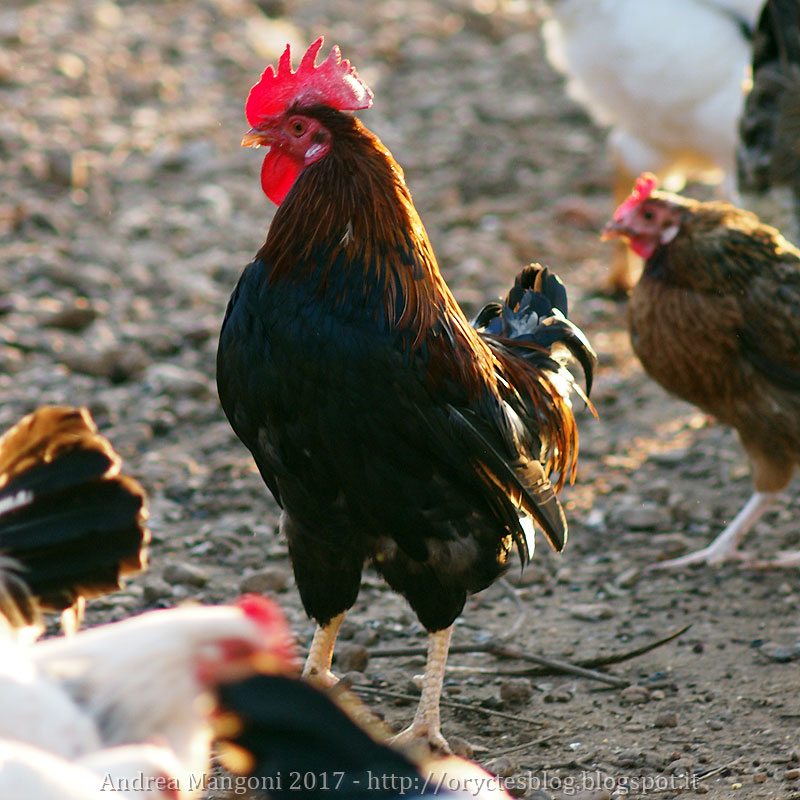 |
| 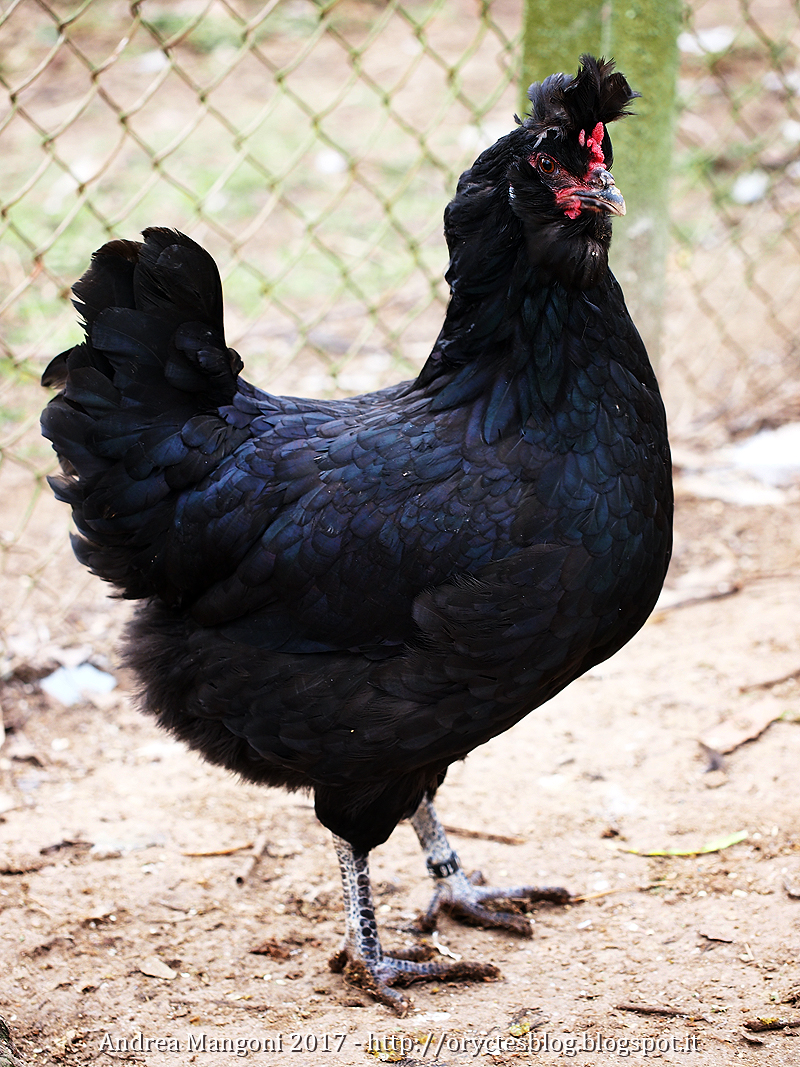 | Polverara Nera  (PPN) | Robusta Lionata  (PRL) | 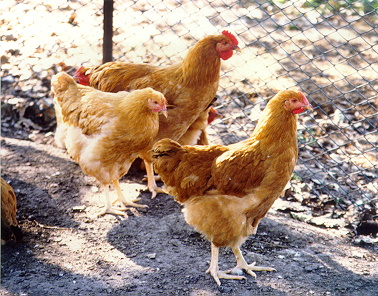 |
| 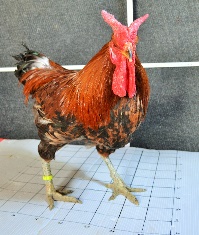 | Cornuta di Caltanissetta  (COR) | Robusta Maculata  (PRM) | 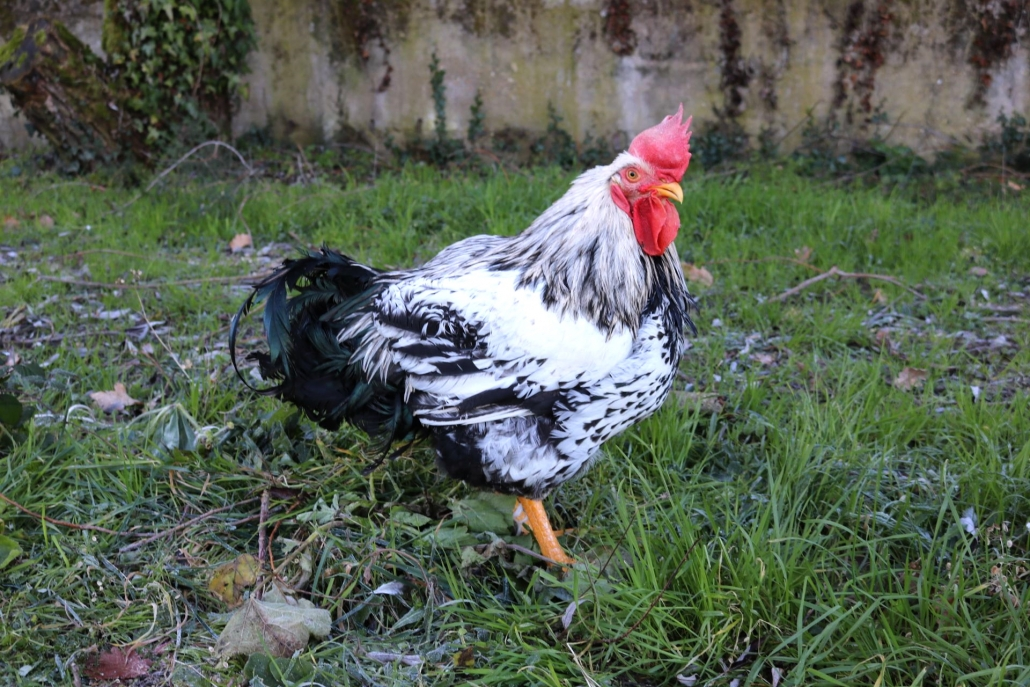 |
| 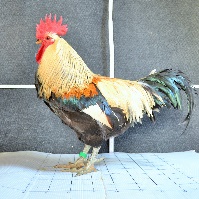 | Valplatani  (VAP) | Valdarnese Bianca  (VAD) | 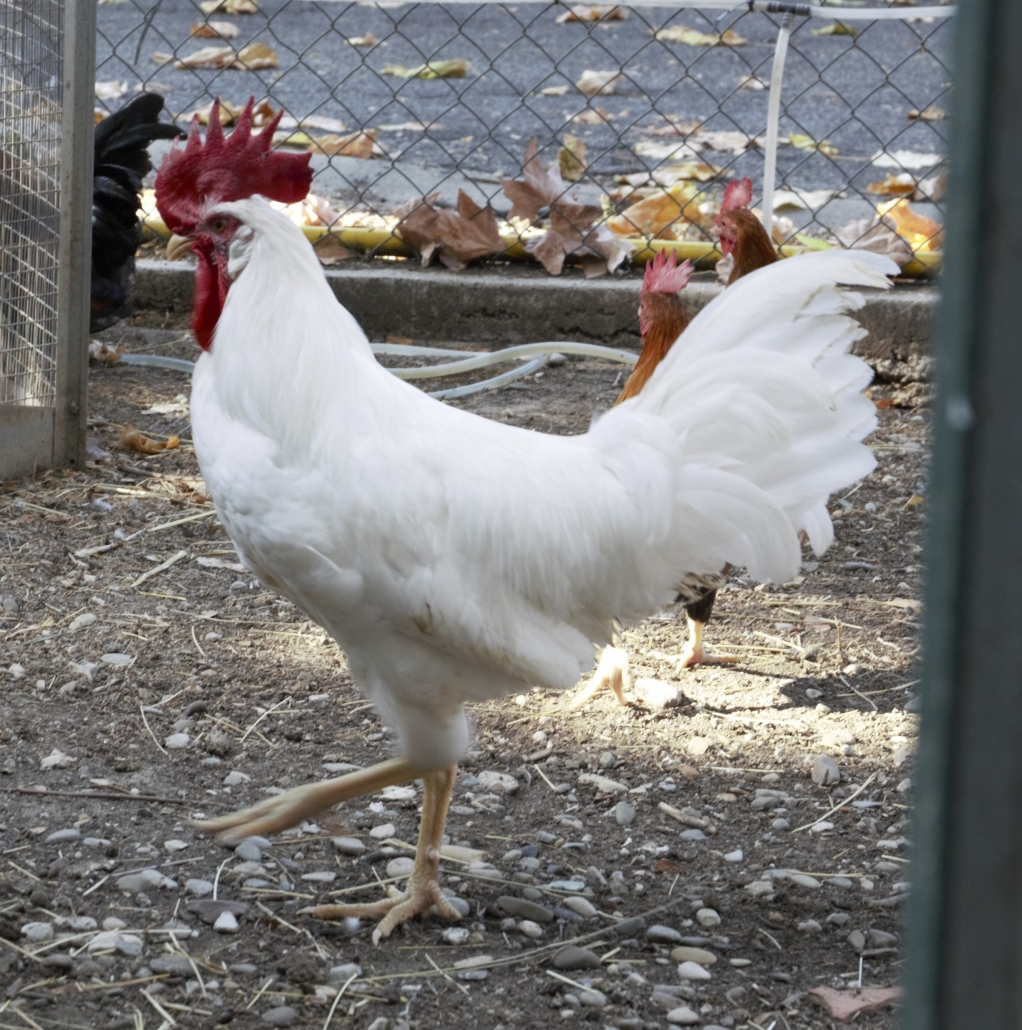 |
| 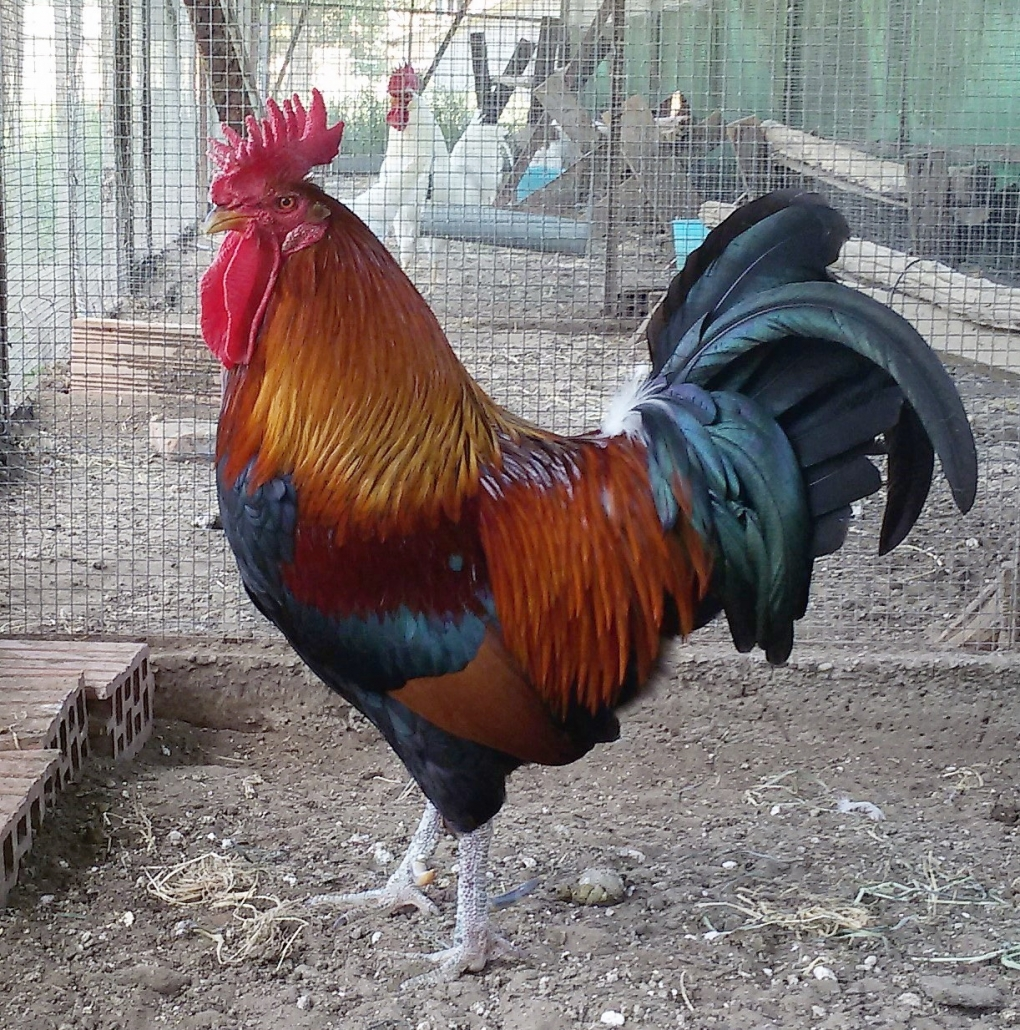 | Siciliana  (SIC) |  |  |
| 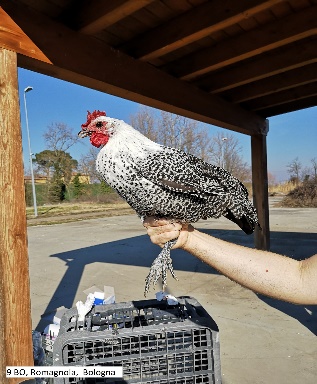 | Romagnola  (ROM) |  |  |

**Supplementary Table 1.** Italian local chicken breeds grouped according to shank pigmentation.
